# Supplementary material for: Febrile Rash: An Early Diagnostic Clue to Infectious Illness in Travelers Returning from Thailand
Source: Reports (MDPI). 2024 Jun 7;7(2):45. doi: 10.3390/reports7020045 (PMC12225320; doi:10.3390/reports7020045)
Supplement: Supplementary file 1 [file reports-07-00045-s001.zip › reports-2893457-supplementary.pdf]

reports

MDPI

Interesting Images

Febrile Rash: An Early Diagnostic Clue to Infectious Illness in Travelers Returning from Thailand

Hisham Ahmed Imad [1,2,3,*], Anastasia Putri [1,2], Ratchata Charoenwisedsil [1], Sakarn Charoensakulchai [1] and Eric Caumes [4]

[1] Thai Travel Clinic, Hospital for Tropical Diseases, Faculty of Tropical Medicine, Mahidol University, Bangkok 10400, Thailand; anastasia@thaitravelclinic.com (A.P.); rachata@thaitravelclinic.com (R.C.); sakarn@thaitravelclinic.com (S.C.)
[2] Department of Clinical Tropical Medicine, Faculty of Tropical Medicine, Mahidol University, Bangkok 10400, Thailand
[3] Center for Infectious Diseases Education and Research, Department of Viral Infections, Research Institute for Microbial Diseases, Osaka University, Osaka 565-0871, Japan
[4] Centre de Diagnostic et de Thérapeutique, Hotel Dieu Hôpital, 75004 Paris, France; eric.caumes@aphp.fr
* Correspondence: hishamahmed.ima@mahidol.ac.th

Table S1. Serial hematological profile and other investigations

Days after onset of symptoms	Day 1	Day 2	Day 3	Day 4	Day 5	Day 6	Day 7	Day 24‡
Hemoglobin, g/dL (32–36)	16.5	15.3	15.1	16.3	15.6	15.6	16.2	15.5
Hematocrit, % (40–54)	48.9	45.5	45.1	48.4	46.4	46.6	47.1	48.6
Leukocytes, cells/μL (5–10)	4500	3900	2900	2400	2500	5800	5600	3000
Neutrophils, % (40–74)	77	72	73	73	25	32	35	45
Lymphocytes, % (16–45)	15	11	15	15	38	26	34	46
Monocytes, % (0–10)	5	6	6	6	9	4	5	2
Eosinophils, % (0–10)	0	0	2	2	0	1	6	2
Basophils, % (0–2)	0	0	0	0	1	0	0	0
Platelets, cells/μL (150,000–450,000)	177,00	154,000	128,000	107,000	68,000	72,000	122,000	227,000
Serum Creatinine, mg/dL (0.67-1.17)	1.3		1.04					
Aspartate aminotransferase, U/L (0-40)	34		35				72	46
Alanine aminotransferase, U/L (0-41)	55		51				72	98
Dengue NS1	negative				negative			
anti-dengue IgM	negative				positive			
anti-dengue IgG	negative				positive			
anti-chikungunya IgM	negative							
anti-chikungunya IgG	negative							

‡ Follow up visit

Disclaimer/Publisher's Note: The statements, opinions and data contained in all publications are solely those of the individual author(s) and contributor(s) and not of MDPI and/or the editor(s). MDPI and/or the editor(s) disclaim responsibility for any injury to people or property resulting from any ideas, methods, instructions or products referred to in the content.
